# NucVoter: A Voting Algorithm for Reliable Nucleosome Prediction Using Next-Generation Sequencing Data

**DOI:** 10.1155/2013/174064

**Published:** 2013-11-07

**Authors:** Boseon Byeon

**Affiliations:** Institute of Molecular Medicine and Genetics, Georgia Regents University, Augusta, GA 30912, USA

## Abstract

Nucleosomes, which consist of DNA wrapped around histone octamers, are dynamic, and their structure, including their location, size, and occupancy, can be transformed. Nucleosomes can regulate gene expression by controlling the DNA accessibility of proteins. Using next-generation sequencing techniques along with such laboratory methods as micrococcal nuclease digestion, predicting the genomic locations of nucleosomes is possible. However, the true locations of nucleosomes are unknown, and it is difficult to determine their exact locations using next-generation sequencing data. This paper proposes a novel voting algorithm, NucVoter, for the reliable prediction of nucleosome locations. Multiple models verify the consensus areas in which nucleosomes are placed by the model with the highest priority. NucVoter significantly improves the performance of nucleosome prediction.

## 1. Introduction

**Figure 1:**
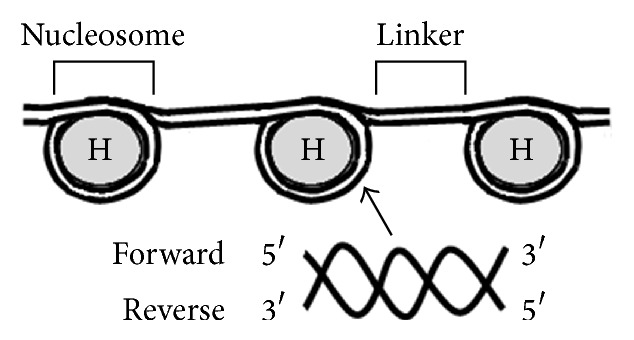
Organization of nucleosomes and linkers, and DNA. A nucleosome is composed of DNA wrapped around a histone octamer. H indicates a histone octamer. Nucleosomes are connected by linker DNA. DNA is double stranded; the forward strand is in the 5′ to 3′ direction, while the reverse strand is in the opposite direction.

Genes within DNA are transcribed into an RNA product [1]. To be transcribed, the DNA region encoding a gene must be accessible to proteins such as transcription factors and RNA polymerase [2]. As shown in Figure 1, a nucleosome is composed of a DNA sequence wrapped 1.65 times around a histone octamer [3]. If the DNA region is wrapped compactly to prevent proteins from binding to the DNA, the corresponding gene is not transcribed [4]. Therefore, nucleosomes can regulate gene expression by restricting or facilitating the DNA accessibility of proteins.

**Figure 2:**
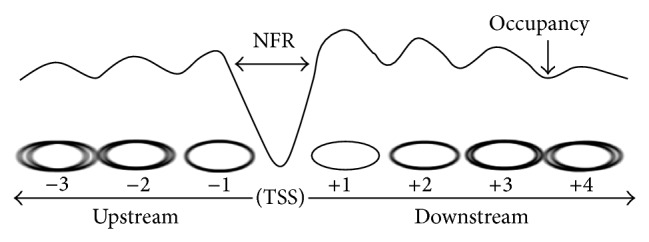
Profile of typical nucleosomes around TSSs of yeast genes.

Figure 2 shows the profile of typical nucleosomes around the transcription start sites (TSSs) of yeast genes. The most prevalent size of nucleosomes is approximately 147 base pairs (bp), and the length of linker DNA between nucleosomes is approximately 18 bp [3]. The occupancy of a nucleosome represents the possibility that a nucleosome resides at a particular genomic location. The so-called −1 nucleosome is the first nucleosome upstream of the TSS. The area downstream of the −1 nucleosome is the nucleosome-free region (NFR) which shows very low nucleosome occupancies over approximately 150 bp on average [5]. The NFR contains transcription factor binding sites and is therefore important in transcription regulation [6]. The first nucleosome downstream of the NFR is the +1 nucleosome, followed by the +2, +3, and +4 nucleosomes. While the +1 nucleosome is stable, its upstream and downstream nucleosomes show declines in occupancy and stability and become fuzzy.

For proteins to bind the DNA region associated with compact nucleosomes to initiate transcription, the nucleosome structure needs to be changed. Interestingly, nucleosomes are dynamic, and their structure can be transformed [6, 7]. ATP-dependent remodeling can slide histone octamers a short distance along DNA or remove them temporarily from DNA. Additionally, chemical modifications of histones or histone replacement with histone variants can alter the structure of nucleosomes.

**Figure 3:**
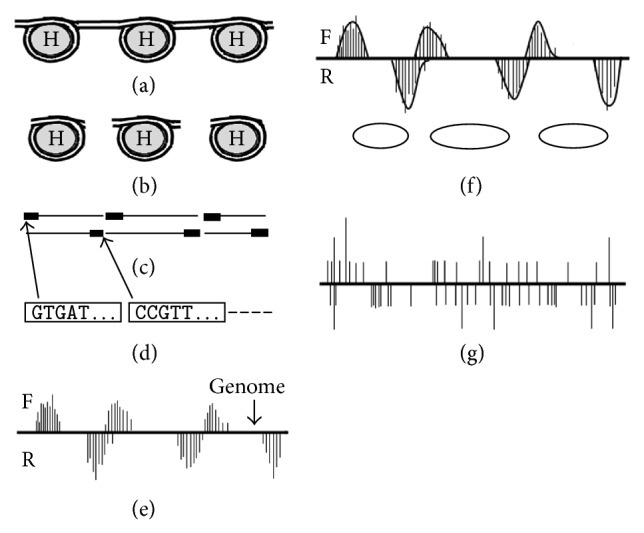
Nucleosome prediction process. (a) Nucleosomes connected by linker DNA. (b) Nucleosomes following MNase digestion. (c) Purified DNA fragments. Arrowed marks indicate the 5′ ends, which are sequenced via NGS. (d) Short sequence tags generated by NGS. (e) Distribution of tags mapped to the reference genome. F and R indicate the forward and reverse strands, respectively. (f) Tag smoothing and nucleosome prediction. Ellipses represent predicted nucleosomes. (g) Example of tag distributions, which are not well separated.

Nucleosome prediction refers to nucleosome positioning in the genome. Using MNase-seq or Chip-seq, it is possible to identify the genomic locations of nucleosomes [4, 5, 8]. Micrococcal nuclease (MNase) is a restriction enzyme that digests DNA that is not wrapped around histones. By treating cells with MNase, linker DNA is removed, and nucleosomes can be extracted (Figures 3(a) and 3(b)). Immunoprecipitation (Chip) with histone antibodies can be further employed to select nucleosomes with regard to specific histone modifications. DNA fragments are subsequently purified from the nucleosomes (Figure 3(c)). Next-generation sequencing (NGS) technologies generate short sequences (25–36 bp), referred to as tags, from the 5′ ends of purified DNA fragments in a cost-effective manner [9, 10] (Figure 3(d)). As shown in Figure 1, DNA is double stranded, with the forward strand oriented in the 5′ to 3′ direction and the reverse strand in the opposite direction. Therefore, the sequence tags from the 5′ ends of the forward and reverse strands represent the left and right boundaries of nucleosomes, respectively (Figure 3(c)).

To determine the genomic locations of nucleosomes, the sequence tags are mapped to a reference genome. Nucleosome locations differ from cell to cell, and MNase digests DNA at various levels. Therefore, mapping short sequences to a reference genome produces a series of distributions with regard to the frequencies of tags starting at each genomic location (Figure 3(e)). A simple algorithm to predict the positions of nucleosomes along the genome smooths the tag distributions and positions nucleosomes with their left and right boundaries at the local maxima of the distributions on the forward and reverse strands, respectively (Figure 3(f)). The occupancy of a nucleosome is derived from the frequencies of tags that are used to position the nucleosome. Many methods for nucleosome prediction are variants of this algorithm [11–14].

However, the true locations of nucleosomes are unknown because MNase does not digest linker DNA precisely [5]. Furthermore, tag distributions are not well separated in many genomic regions (Figure 3(g)). Therefore, nucleosome prediction using next-generation sequencing data is difficult. In the next section, nucleosome positioning methods are introduced, and a novel voting algorithm is proposed for reliable nucleosome prediction.

## 2. Methodology

### 2.1. Nucleosome Prediction Methods

GeneTrack shifts sequence tags toward the 3′ direction by half of the user-defined nucleosome size, smooths the tag distributions using a Gaussian smoothing procedure, and then positions nucleosomes by setting the local maxima to the centers of nucleosome [15]. Nucleosomes can be defined separately on the forward and reverse strands or on the composite strand derived from the sum of the tag frequencies on the forward and reverse strands.

NSeq generates probabilistic distributions of nucleosome centers based on sequence tags and determines significant nucleosomes using triangle statistics, N statistics, and false discovery rates [16]. Nucleosomes predicted by NSeq also have a predetermined size.

TemplateFilter uses templates representing diverse patterns of distributions [17]. TemplateFilter identifies the genomic locations where its templates, including a normal-shaped template, correlate with tag distributions. Then, the locations of optimal templates correlated with the forward and reverse tag distributions determine the left and right boundaries of nucleosomes, respectively. Therefore, TemplateFilter defines nucleosomes of various sizes differently from GeneTrack and NSeq.

### 2.2. Voting Algorithm

In machine learning, one approach for finding a reliable solution to a difficult problem is the ensemble method, which combines the outcomes of different models [18]. The simplest ensemble technique is voting, in which multiple models take votes, and the majority outcome is adopted as the solution.

**Figure 4:**
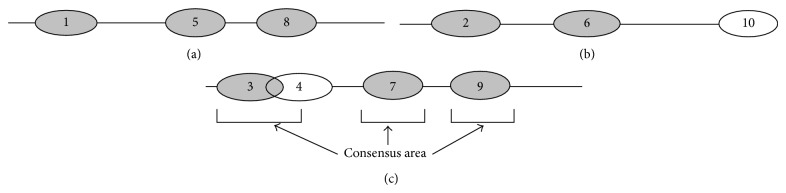
Consensus nucleosomes and consensus areas. (a), (b), and (c) indicate three different prediction methods. Grey ellipses represent consensus nucleosomes. Nucleosomes 6 and 7, which are globally nearest, initially create a consensus area, and nucleosome 5 is added. Then, nucleosomes 8 and 9, which are nearest among the remaining nucleosomes, create another consensus area. Finally, nucleosomes 1 and 2 create a consensus area, and then nucleosome 3 is added. Adding nucleosome 3 rather than nucleosome 4 minimizes the distances of the centers of the three nucleosomes in the consensus area. Under the assumption that the priorities are in the order of methods (b), (a), and (c), NucVoter predicts nucleosomes 2, 6, and 8.

In NucVoter, a nucleosome prediction method is regarded as a voter, and the three voters described above locate nucleosomes across the genome. If two or more of the voters position nucleosomes around a genomic location, NucVoter defines those nucleosomes as consensus nucleosomes and their nucleosomal locations as consensus areas where true nucleosomes are likely to reside (Figure 4). Then, NucVoter assigns a priority to each voter as described below and chooses the nucleosome predicted by the voter with the highest priority in each consensus area. If only one voter places a nucleosome in a particular genomic location, NucVoter concludes that the site does not contain a nucleosome.

In a preliminary process, NucVoter normalizes the occupancies of nucleosomes from each voter so that the values show a zero mean and 1 standard deviation. Then, NucVoter further makes the entire occupancies positive by subtracting the global minimum occupancy, which is a negative value, from the normalized occupancies.

To establish the priority of each voter, NucVoter uses the consensus nucleosomes receiving the consent of all three voters. Then, it is hypothesized that if the consensus nucleosomes in a consensus area are nearer, they display higher occupancies than those in other consensus areas, meaning that if voters predict nucleosomes within a short distance of each other, the possibility that a nucleosome resides in that region is high. The consensus nucleosomes with the consent of all three voters are extracted, and their averaged center distance is computed in each consensus area. Then, the correlation between the occupancies of the consensus nucleosomes of each voter and their corresponding averaged center distances is calculated. NucVoter regards the voter showing the higher negative correlation as more accurate and assigns the higher priority to that voter.

Figure 4 depicts the consensus nucleosomes and areas. In NucVoter, the distances between the centers of the consensus nucleosomes in a consensus area should be ≤73 bp (half of the prevalent nucleosome size). The two globally nearest nucleosomes predicted by two different voters create a consensus area, and the third consensus nucleosome predicted by the other voter is added so that the distances between the centers of all consensus nucleosomes are minimized. This process is iterated among the remaining nucleosomes until no more consensus nucleosomes exist. Then, NucVoter determines the priority of each voter using the consensus nucleosomes receiving the consent of all three voters, as described above, and chooses the nucleosome predicted by the voter with the highest priority in each consensus area where two or more voters consented. 

When two or more datasets need to be compared (see Section 3.2), NucVoter sets the global voting priorities on the basis of the averaged correlation of each voter across the datasets. Then, the global priorities are used to choose nucleosomes consistently in all datasets.

### 2.3. Software Availability

NucVoter is available on request.

## 3. Results

### 3.1. Synthetic Data

Because the exact locations of nucleosomes are unknown, synthetic datasets were generated to measure the performance of NucVoter. The data generation procedure was carried out in a manner similar to the function of “syntheticNucMap” embedded in the R package of nucleR [19].

A total of 1000 stable nucleosomes were generated periodically on the basis of a nucleosome size of 146 bp and a linker length of 20 bp, and 50 nucleosomes were randomly removed. Then, the F number of fuzzy nucleosomes was added at random. The forward/reverse tags for both stable and fuzzy nucleosomes were randomly generated in the range of 1 to the C coverage (i.e., the number of tags) at the starting/ending locations of nucleosomes. Then, stable tags were randomly shifted in the range of +/−20 bp and fuzzy tags in the range of +/−50 bp. Finally, 49000 true nucleosomes in 40 synthetic datasets were generated based on the combination of various numbers of fuzzy nucleosomes (F: 50 to 500 with an interval of 50) and different coverage (C: 50 to 200 with an interval of 50). Note that most of the parameter values used for this process were the default values of the “syntheticNucMap” function.

GeneTrack, with a nucleosome size 146 bp on the composite strand, as well as NSeq and TemplateFilter, was initially executed using their default parameter values to predict synthetic nucleosomes. However, NSeq and TemplateFilter positioned too few nucleosomes. Therefore, to generate a reasonable number of nucleosomes, the “-f  1” option was used for NSeq, and the options “-overlap 1.0” and “-corr_bound 0.3” were used for TemplateFilter. Note that the parameter values were not optimized because the goal of implementation was not to compare the performance of existing methods but to examine how the proposed voting algorithm improves the prediction capability given the outputs of individual methods.

Table 1 shows the prediction of true nucleosomes in the synthetic datasets. If the distance between the centers of a true nucleosome and the nearest predicted nucleosome is ≤73 bp, the true nucleosome is regarded as correctly predicted. The true positives indicate the correctly positioned true nucleosomes, and the false positives represent the incorrectly positioned false nucleosomes. The false negatives are the missed true nucleosomes. Although TemplateFilter predicted the greatest number of true positives, it also positioned many false positives. NSeq placed the least false positives but missed many true positives. NucVoter and GeneTrack showed reasonably good performance.

**Figure 5:**
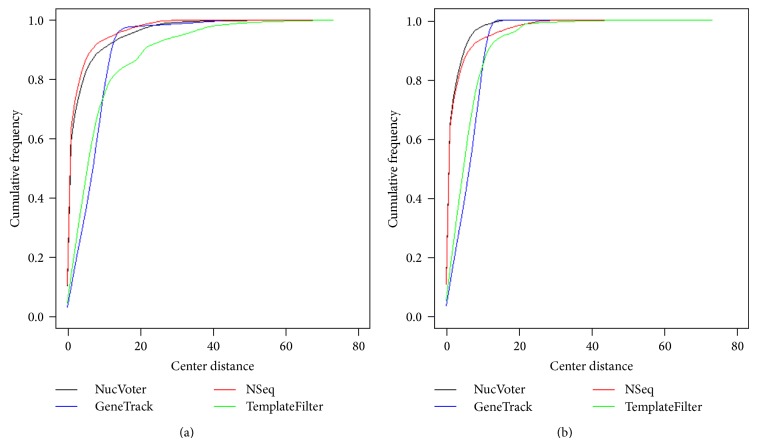
Cumulative frequency ratio as the function of the distance between the centers of the predicted and true nucleosomes. (a) All true positives were used to generate the plot. Therefore, the number of nucleosomes across the indicated methods is different. (b) The same number of the true positives nearest to the true nucleosomes was extracted from each dataset and used to generate the plot.

Using the true positives, the cumulative frequency ratio was plotted as a function of the distance between the centers of the true and predicted nucleosomes (Figure 5(a)). While NucVoter and NSeq predicted nucleosomes accurately, GeneTrack and TemplateFilter did so less accurately. However, these results might have been produced because different numbers of nucleosomes were used for plotting. Thus, the same number of the true positives nearest to the true nucleosomes was extracted from each dataset, and the plot of their cumulative frequency was drawn (Figure 5(b)). As expected, the performances of GeneTrack and TemplateFilter were higher. Furthermore, NucVoter outperformed the other methods significantly (*P* values of the Kolmogorov-Smirnov test < 2.2*e* − 16). The centers of 90% of the NucVoter nucleosomes were within 5 bp from the centers of true nucleosomes. This result supports the hypothesis that the proposed voting algorithm improves the accuracy of nucleosome prediction.

**Figure 6:**
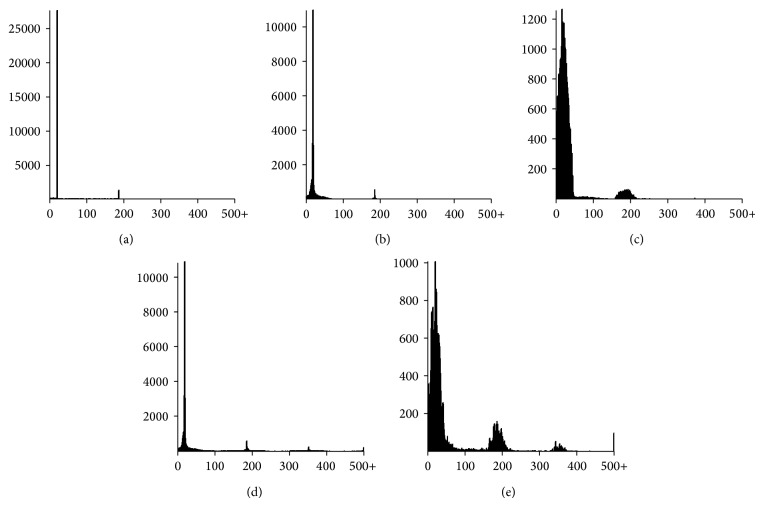
Frequency of linker lengths. (a) Frequency of the lengths of true linkers. Frequencies of the lengths of linkers predicted by (b) NucVoter, (c) GeneTrack, (d) NSeq, and (e) TemplateFilter.

To further analyze the prediction improvement achieved by NucVoter, linker analysis was performed.  Figure 6 shows the linker length frequencies obtained. As noted above, the length of true linkers in the synthetic data was 20 bp, and the plot therefore displays a particularly high frequency peak at 20 bp (Figure 6(a)). Additionally, because 5% of nucleosomes were randomly removed, there was a high frequency peak at 186 bp (i.e., 146 bp + 20 bp + 20 bp). Although there were various linker lengths of less than 186 bp observed due to the presence of fuzzy nucleosomes, there were no linkers longer than 186 bp. In the region below 200 bp, NucVoter and NSeq produced frequencies that were more similar to the real linker frequency compared to GeneTrack and TemplateFilter (Figures 6(b) to 6(e)), which means that NucVoter and NSeq predicted nucleosomes more accurately. However, NSeq and TemplateFilter exhibited relatively high frequencies, approximately 352 bp and in the area of 500 bp or longer (Figures 6(d) and 6(e)), which indicated missing consecutive true nucleosomes.

To measure the overall performance of NucVoter, individual locations in synthetic genomes were scanned, and a confusion matrix of the locations of nucleosomal and linker DNA was generated [18, 20]. True positives (TP) and true negatives (TN) represent correct predictions of nucleosomal and linker locations, respectively. While a false positive (FP) occurs when a linker location is incorrectly predicted as a nucleosomal location, a false negative (FN) is observed when a nucleosomal location is incorrectly predicted as a linker location. The sensitivity is TP divided by TP + FN and measures the ability to correctly predict true nucleosomal locations. The specificity, which is TN divided by TN + FP, measures the ability to correctly predict true linker locations. The overall accuracy is defined as the number of correct predictions divided by the total number of predictions (i.e., TP + TN over TP + TN + FP + FN). As observed in Table 2, NucVoter achieved the best performance in terms of both sensitivity and overall accuracy. Regarding specificity, NucVoter displayed a performance similar to that of NSeq, which exhibited the best performance. These results confirm that the proposed voting algorithm significantly improves the nucleosome prediction capability.

### 3.2. Real Data

NucVoter was applied to published MNase-seq datasets generated from normal and heat-shocked yeast cells [14]. To achieve consistent voting between the two datasets, the global priorities for nucleosome selection were set in the order of TemplateFilter, NSeq, and GeneTrack on the basis of the averaged correlation values across the two datasets, as described above. To make the occupancy levels of two datasets comparable, the occupancy values of nucleosomes predicted by NucVoter were further normalized via the quantile method.

**Figure 7:**
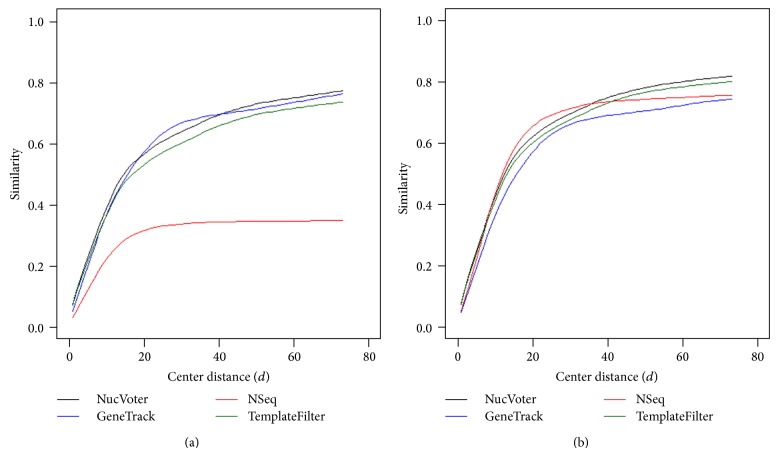
Similarity as a function of the distance between the centers of the published nucleosomes and the nucleosomes predicted by the indicated methods.

Additionally, GeneTrack and NSeq were executed with the “-f  1” option, and TemplateFilter was run with its default options to generate a reasonable number of nucleosomes.  Figure 7 shows the similarity of the predicted nucleosomes to published nucleosomes. Assuming the published nucleosomes to be true nucleosomes, if the distance between the centers of a published nucleosome and the nearest predicted nucleosome was ≤*d*, the published nucleosome was regarded as correctly predicted. Then, the similarity was defined as the number of correctly predicted nucleosomes divided by the total number of predicted nucleosomes (i.e., TP over TP + FP + FN). Refer to Table 1 description for the definitions of TP, FP, and FN. Interestingly the nucleosomes predicted by NucVoter showed the highest similarity to the published nucleosomes overall.

**Figure 8:**
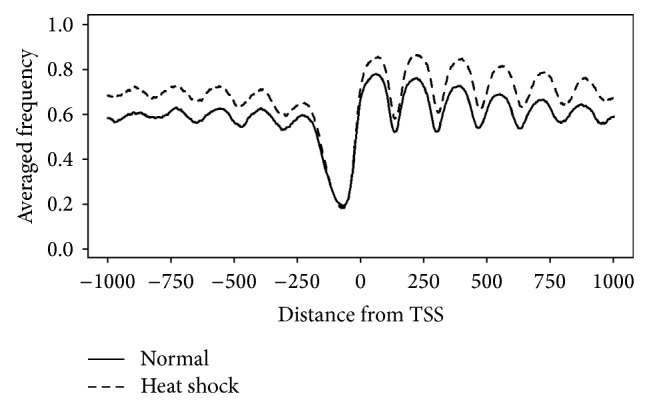
Averaged frequency of the nucleosomes predicted by NucVoter as a function of the distance from the TSSs of yeast genes.

Figure 8 depicts the averaged frequency of nucleosomes predicted by NucVoter as a function of the distance from TSSs. The number of nucleosomes in the heat-shocked cells is greater than the number in normal cells, which indicates that nucleosomes are gained under heat shock. While the frequencies of the nucleosomes downstream of the +1 nucleosome decrease slightly, the numbers of the nucleosomes upstream of the −1 nucleosome increase to a small degree.

**Figure 9:**
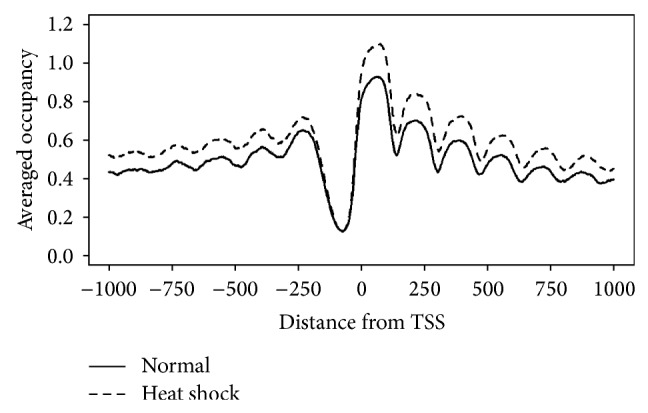
Averaged occupancy of the nucleosomes predicted by NucVoter as a function of the distance from the TSSs of yeast genes.

Figure 9 is the averaged occupancy plot for the nucleosomes predicted by NucVoter as a function of the distance from TSSs. It can be observed that the occupancy of nucleosomes in heat-shocked cells is higher than in normal cells. Note that this is identical to the published result [14]. The +1 nucleosome displays strong occupancy, and its downstream nucleosomes show substantially decreased occupancy. Although the −1 nucleosome exhibits a lower frequency than its upstream nucleosomes, as shown in Figure 8, its occupancy is higher. Additionally, the length of the NFR is slightly shorter in the heat-shocked compared to the normal cells.

**Figure 10:**
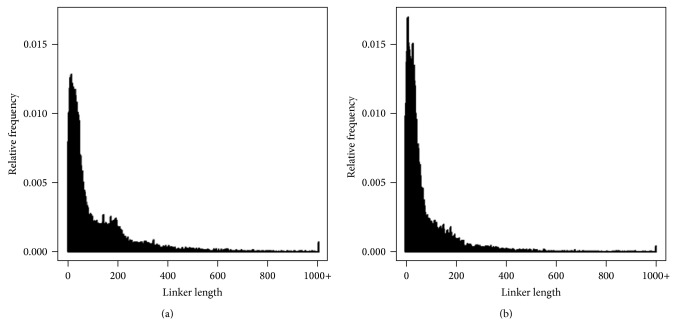
Relative frequency of the lengths of linkers predicted by NucVoter in the range of +/−1000 bp from the TSSs of yeast genes. (a) Linkers from normal cells. (b) Linkers from heat-shocked cells.

Figures 10(a) and 10(b) provide plots of the frequencies of the lengths of linkers predicted by NucVoter in the range of +/−1000 bp from the TSSs of genes. The linkers are shorter overall in the heat-shocked cells than in the normal cells, and the small peak observed at approximately 180 bp in the normal cells, which reflects the NFR, disappears following heat shock.

**Figure 11:**
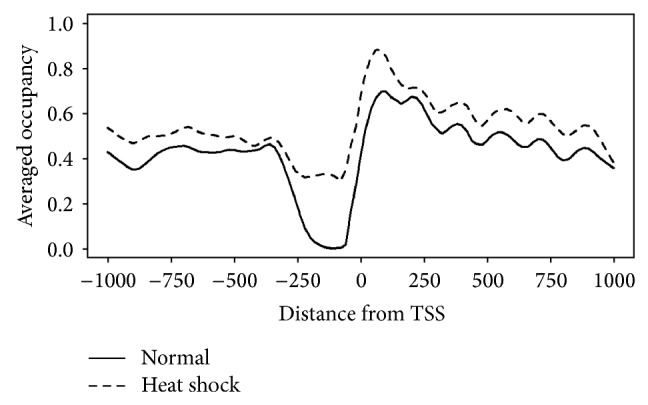
The averaged occupancy of the nucleosomes predicted by NucVoter as a function of the distance from the TSSs of the 264 yeast genes whose NFR length was reduced by ≥150 bp following heat shock. The plot was smoothed by Lowess method [2].

To analyze the NFRs predicted by NucVoter, the longest linkers in the range of TSS − 250 bp to TSS + 50 bp were defined as NFRs, and the 264 genes whose NFR lengths were reduced by ≥150 bp following heat shock were extracted. Figure 11 provides the nucleosome profile of these genes aligned based on their TSSs. Then, Gene Ontology (GO) analysis of the genes was performed using FunSpec [21, 22]. GO provides annotation of genes based on biological knowledge [2].  Table 3 describes the GO biological processes of the 264 genes. A total of 29 and 28 genes significantly contribute to translation and metabolic processes, respectively. This result supports the notion that the NFR plays an important role in regulating gene expression.

## 4. Conclusions

Although nucleosomes are critical for regulating gene expression, the prediction of nucleosome locations is difficult. This paper proposed the NucVoter algorithm, which is a novel voting algorithm for reliable nucleosome prediction using next-generation sequencing data.

Synthetic datasets were generated and employed to demonstrate that NucVoter significantly improved the accuracy of nucleosome prediction because the locations of true nucleosomes were unknown. NucVoter predicted nucleosomes closer to true nucleosomes than any other method examined in this analysis. In addition, NucVoter produced linkers in a manner that was more similar to actual linkers than most of the other methods. Furthermore, it was confirmed that NucVoter achieved the best prediction accuracy and significantly improved the performance of nucleosome prediction.

Using published datasets from normal and heat-shocked yeast cells, it was shown that NucVoter could be applied to various nucleosome analyses. Many nucleosomes were observed to be gained under heat shock, and the occupancy of nucleosomes in the heat-shocked cells was higher than in normal cells. Additionally, the linkers became shorter in the heat-shocked cells compared to the normal cells. Based on GO analysis, it was further noted that genes whose NFR lengths were considerably reduced following heat shock contributed to translation and metabolic processes.

## Figures and Tables

**Table 1:** Number of synthetic nucleosomes predicted by the indicated methods.

Method	True positives	False positives	False negatives
NucVoter	38217	34	10783
GeneTrack	38706	13	10294
NSeq	35114	10	13886
TemplateFilter	39490	1710	9510

**Table 2:** Accuracy of the nucleosomal and linker locations predicted by the indicated methods. The number in parentheses is the *P* value of the paired  *t*-test, where the alternative hypothesis is that the accuracy of NucVoter is greater than that of the indicated method.

Method	Sensitivity	Specificity	Overall accuracy
NucVoter	94.25%	94.00%	94.22%
GeneTrack	92.95% (5.254*e* − 09)	75.34% (<2.2*e* − 16)	90.73% (<2.2*e* − 16)
NSeq	87.89% (6.514*e* − 12)	94.61% (1.0)	88.73% (1.117*e* − 11)
TemplateFilter	84.12% (<2.2*e* − 16)	79.58% (<2.2*e* − 16)	83.55% (<2.2*e* − 16)

**Table 3:** GO analysis of the 264 genes whose NFR length was reduced by ≥150 bp following heat shock.

Biological process	*P* value	Number of genes
Translation [GO:0006412]	2.31*e* − 05	29
Nucleobase, nucleoside, nucleotide, and nucleic acid transport [GO:0015931]	0.000660	4
Tryptophan transport [GO:0015827]	0.001593	2
Ergosterol biosynthetic process [GO:0006696]	0.001830	5
Steroid biosynthetic process [GO:0006694]	0.002706	5
Metabolic process [GO:0008152]	0.005827	28
Nucleobase transport [GO:0015851]	0.006152	3
Group I intron splicing [GO:0000372]	0.006152	3
